# Reshaping the tumor microenvironment of cold soft-tissue sarcomas with oncolytic viral therapy: a phase 2 trial of intratumoral JX-594 combined with avelumab and low-dose cyclophosphamide

**DOI:** 10.1186/s12943-024-01946-8

**Published:** 2024-02-20

**Authors:** Maud Toulmonde, Jean-Philippe Guegan, Mariella Spalato-Ceruso, Florent Peyraud, Michèle Kind, Lucile Vanhersecke, François Le Loarer, Raul Perret, Coralie Cantarel, Carine Bellera, Alban Bessede, Antoine Italiano

**Affiliations:** 1 Early Phase Trials and Sarcoma Units, Department of Medical Oncology, Institut Bergonié, 229 Cours de l’Argonne, Bordeaux, France; 2 Explicyte, Bordeaux, France; 3 Department of Medical Imaging, Institut Bergonié, Bordeaux, France; 4 Department of Pathology, Institut Bergonié, Bordeaux, France; 5 Faculty of Medicine, University of Bordeaux, Bordeaux, France; 6 Bordeaux Population Health Research Center, Univ. Bordeaux, Epicene team, UMR 1219, Inserm, Bordeaux, F-33000 France; 7 Clinical and Epidemiological Research Unit, Comprehensive Cancer Center, Inserm CIC1401, Institut Bergonié, Bordeaux, F-33000 France

**Keywords:** Soft-tissue sarcoma, Oncolytic virus, JX-594, Low-dose cyclophosphamide, Avelumab

## Abstract

Most soft-tissue sarcomas (STS) exhibit an immunosuppressive tumor microenvironment (TME), leading to resistance against immune checkpoint inhibitors (ICIs) and limited therapeutic response. Preclinical data suggest that oncolytic viral therapy can remodel the TME, facilitating T cell accumulation and enhancing the immunogenicity of these tumors.

We conducted the METROMAJX, a phase II clinical trial, to investigate the combination of JX-594, an oncolytic vaccinia virus engineered for selective tumor cell replication, with metronomic cyclophosphamide and the PD-L1 inhibitor avelumab in patients with advanced, ‘cold’ STS, characterized by an absence of tertiary lymphoid structures. The trial employed a two-stage Simon design. JX-594 was administered intratumorally at a dose of 1.10^9^ pfu every 2 weeks for up to 4 intra-tumoral administrations. Cyclophosphamide was given orally at 50 mg twice daily in a week-on, week-off schedule, and avelumab was administered at 10 mg/kg biweekly. The primary endpoint was the 6-month non-progression rate.

Fifteen patients were enrolled, with the most frequent toxicities being grade 1 fatigue and fever. Fourteen patients were assessable for efficacy analysis. At 6 months, only one patient remained progression-free, indicating that the trial did not meet the first stage endpoint of Simon’s design. Analysis of sequential tissue biopsies and plasma samples revealed an increase in CD8 density and upregulation of immune-related protein biomarkers, including CXCL10.

Intra-tumoral administration of JX-594 in combination with cyclophosphamide and avelumab is safe and capable of modulating the TME in cold STS. However, the limited efficacy observed warrants further research to define the therapeutic potential of oncolytic viruses, particularly in relation to specific histological subtypes of STS.

**Supplementary Information:**

The online version contains supplementary material available at 10.1186/s12943-024-01946-8.

## Introduction

The current approach to treating advanced or metastatic soft-tissue sarcomas (STS) primarily involves traditional chemotherapy, which often shows limited effectiveness. This highlights the pressing need for novel therapeutic strategies, among which immunotherapy has shown groundbreaking success in various tumor types.

Petitprez et al. [1] conducted a comprehensive analysis of the STS microenvironment, proposing an immune classification based on transcriptomic data. This classification outlines five sarcoma immune classes (SIC), each characterized by different levels of immune infiltration. A key discovery was the presence of tertiary lymphoid structures (TLS) in the immune-high class, which are associated with improved outcomes and predictive of immunotherapy response. TLS are ectopic lymphoid formations resembling lymph nodes, comprising B-cell follicles, germinal centers, and a T-cell area. They play a vital role in anti-tumor immunity by facilitating immune cell interactions and enhancing local immune responses [2]. In the first biomarker-driven immunotherapy trial for STS, the presence of TLS correlated with better outcomes and increased sensitivity to immune checkpoint inhibitors, with TLS-positive STS patients showing a 30% objective response rate [3].

These findings suggest that immunotherapy (IT) could be a more effective treatment for certain sarcoma patients compared to conventional cytotoxic chemotherapy (CCT). However, the challenge lies in extending these benefits to the 80% of patients with TLS-negative sarcomas. In addressing this, strategies to convert ‘cold’ tumors into ‘hot’, immune-responsive states needs to be explored.

Oncolytic viruses (OV) administration triggers a strong antiviral immune response, releasing cytokines such as type-1 interferons and chemokines, which attract immune cells [4]. This process effectively transforms the tumor environment, making it more sensitive to checkpoint inhibitors [5].

Additionally, certain chemotherapy regimens, like metronomic chemotherapy (MC), have shown immunomodulatory effects. MC, defined as the regular administration of low-dose cytotoxic agents [6], has both antiangiogenic and immunomodulatory properties, making it a multifaceted therapy. It’s notably used in treating STS, with some efficacy demonstrated in studies [7].

JX-594 (Pexa-Vec; Jennerex Inc.), a thymidine kinase (TK) gene-inactivated OV expressing granulocyte-macrophage colony-stimulating factor (GM-CSF), selectively targets cancer cells and tumor-associated endothelial cells, showing promising safety and efficacy [8, 9]. Its selectivity for cells with TK gene upregulation suggests potential effectiveness in STS, where pRB/E2F pathway deregulation is common [10]. Additionally, metronomic cyclophosphamide has shown synergistic effects with oncolytic adenovirus [11].

Given these insights, we hypothesize that combining metronomic cyclophosphamide with JX-594 could synergistically sensitize tumors to immune checkpoint inhibition in patients with advanced, cold STS characterized by the absence of TLS. This study reports on the efficacy of this combination, along with the PD-L1 inhibitor avelumab, in TLS-negative STS patients.

## Patients and methods

### Patients

Patients had to be aged 18 years or older and have histologically confirmed metastatic with documented disease progression [as per Response Evaluation Criteria in Solid Tumours (RECIST 1.1)] within 6 months prior to entry into the study. Main eligibility criteria included: Age ≥ 18 years ; ECOG ≤ 1; Life expectancy > 3 months ; Measurable disease according to RECIST v1.1 outside any previously irradiated field; Absence of tertiary lymphoid structures ; At least one injectable lesion that is = 10 mm in diameter in at least 1 dimension, or an aggregate of injectable lesions that measures = 10 mm; At least three weeks since last chemotherapy, immunotherapy or any other pharmacological treatment and/or radiotherapy; Adequate hematological, renal, metabolic and hepatic functions; women of childbearing potential must have a negative serum pregnancy test before study entry. Both women and men must agree to use a medically acceptable method of contraception throughout the treatment period and for six months after discontinuation of treatment; patients receiving any substances that are inhibitors or inducers of CYP450 2B6 are ineligible. All patients signed written informed consent prior to any study specific procedure.

### Study design and treatment

This was a single-arm, phase 2, clinical trial based on Simon’s two-stage design and was conducted in accordance with the Declaration of Helsinki and Good Clinical Practices. Patients received 50 mg of CP orally b.i.d. one week on and one week off, intratumoral injection of JX-594 1.10^9^ pfu every two weeks for up to 4 infusions, and avelumab 10 mg/kg every 2 weeks (28-day cycle). Patients discontinued treatment if one of the following occurred: the patient made the decision to withdraw or there was unacceptable toxicity, disease progression as per RECIST 1.1, undercurrent illness, or changes in the patient’s condition preventing further treatment by the judgement of the investigator. Response assessment was carried out every 6 weeks. The response was determined per RECIST 1.13 guidelines after blinded central imaging review. Toxicities were assessed continuously per Common Terminology Criteria for Adverse Events 4.0.

### Tumor biopsies and immunohistochemistry (IHC)

Sequential tumor biopsies from a non-injected lesion at baseline and on Day 1 of Cycle 2 enabled the assessment of the systemic impact of intratumoral JX-594 injection on the sarcoma microenvironment. Formalin-fixed paraffin-embedded (FFPE) tissues were sectioned to 3.5 μm thickness and sections were stained on a Ventana Discovery platform (Ventana, Roche Diagnostics) with anti-CD8 primary antibody (C8/144B, Dako), followed by chromogenic detection using DAB (Ventana). Slides were counterstained with hematoxylin (Ventana), coverslipped and digitized on a PhenoImager HT (Akoya). CD8 + T density was quantified using Inform tissue analysis software (v2.6).

### TLS screening

FFPE sections were stained on a Ventana Discovery Ultra platform (Ventana, Roche Diagnostics) with a validated panel combining the anti-CD3 (2GV6, Ventana), anti-CD20 (L26, Ventana) and anti-CD23 (SP23, Ventana) primary antibodies. Stainings were performed with the protocol RUO discovery universal according to the manufacturer’s recommendations and bound primary antibodies were detected using either OmniMap anti-Ms or Rb-HRP together with DISCOVERY Green, Purple or DAB chromogen detection kit (Ventana). Slides were counterstained with hematoxylin, coverslipped and digitized on a PhenoImager HT (Akoya). TLS assessment was performed by a pathologist as previously described [3].

### Plasma proteomics

Plasma samples were collected before treatment initiation (Day − 7), at baseline, cycle 1 Day15 (C1D15) and cycle 2 Day 1 (C2D1) during the study. Proteomic analysis was performed by using the Target96 Immuno-Oncology panel from Olink technology as previously described [12]. Heatmap was drawn using Pheatmap R package (1.0.12).

### Statistical analysis

The primary endpoint was 6-month non-progression according to RECIST 1.1 criteria, and a Simon’s two-stage design was used. This was computed as the number of patients alive and progression-free at six months, divided by the number of patients included in the population of patients evaluable for efficacy. To be considered in the population of patients evaluable for efficacy, patients had to meet the eligibility criteria and receive at least one dose of CP, one intra-tumoral injection of JX-594 and one infusion of avelumab. As such, patients of the population of patients evaluable for efficacy who died before 6 months were counted in the denominator, but not in the numerator. To distinguish a favorable true non-progression rate of 40% from a null rate of 20% (with 80% power and 5% type I error), 43 eligible and assessable patients were needed. Using 3-month or 6-month PFSs as the principal endpoints for phase 2 trials enrolling patients with STS is an international recommendation from the Sarcoma Tumor Group of the EORTC. Based on these recommendations, a 6-month PFS rate of > or = 30% is considered as a reference value to suggest drug activity in the 1st line setting; for second-line therapy, a 3-month PFS rate of > or = 40% would suggest a drug activity, and < or = 20% would suggest inactivity. Following inclusion of the first 13 assessable patients, accrual could continue for a total of 43 patients if at least four non-progressions were observed. At the end of recruitment, at least thirteen non-progressions were needed to conclude that the investigational treatment had a meaningful effect. Secondary endpoints included the best overall response according to RECIST 1.1 criteria, 1-year PFS, 1-year OS, safety, and correlations with the immunological characteristics of the tumors. PFS was defined as the time from the start of treatment to the time of progression or death (from any cause). OS was defined as the time from the start of treatment to death (from any cause) or the last patient contact. Patients who were alive and progression-free were censored at the date of last follow-up. All enrolled patients who received at least one dose of one of the investigational drugs were eligible for safety analyses.

## Results

Between January 2021 and September 2021, 15 patients with advanced STS were enrolled in the study. One patient was excluded from the efficacy analysis since he refused to complete the first cycle of treatment (Supplementary Fig. 1). Baseline patient characteristics are listed in Table 1. Patients were heavily pre-treated: 9 patients (64.2% of the enrolled population) had received more than two previous lines, with a median number of previous lines of 2 (min:1-max:8) (Table 1).

**Table 1:** Patient characteristics *N* = 14

Variable	N (%)
**Gender, n (%)**	
Male	6 (42.8)
Female	8 (57.2)
**Age**	
Median, years (range)	63 (37–79)
**ECOG PS, n (%)**	
0	4 (28.6)
1	10 (71.4)
**Histological subtype (%)​**	
Leiomyosarcomas	6 (42.8)
Angiosarcomas	2 (14.4)
Other histotypes*	6 (42.8)
**Grade (%)**	
2	5 (35.7)
3	1 (7.1)
Unknow	8 (57.1)
**Stage, n (%)**	
Locally advanced	1 (7.1)
Metastatic	13 (92.9)
**Prior lines of chemotherapy, n (%)​**	
1​	5 (35.7)
2​	1 (7.1)
> 2​	8 (57.1)

*Epithelioid sarcoma *n* = 1, Dedifferentiated liposarcoma *n* = 1, myxoid liposarcoma : *n* = 1, endometrial stroma sarcoma *n* = 1, low grade fibromyxoid sarcoma *n* = 1, Undifferentiated pleomorphic sarcoma *n* = 1

**Fig. 1:**
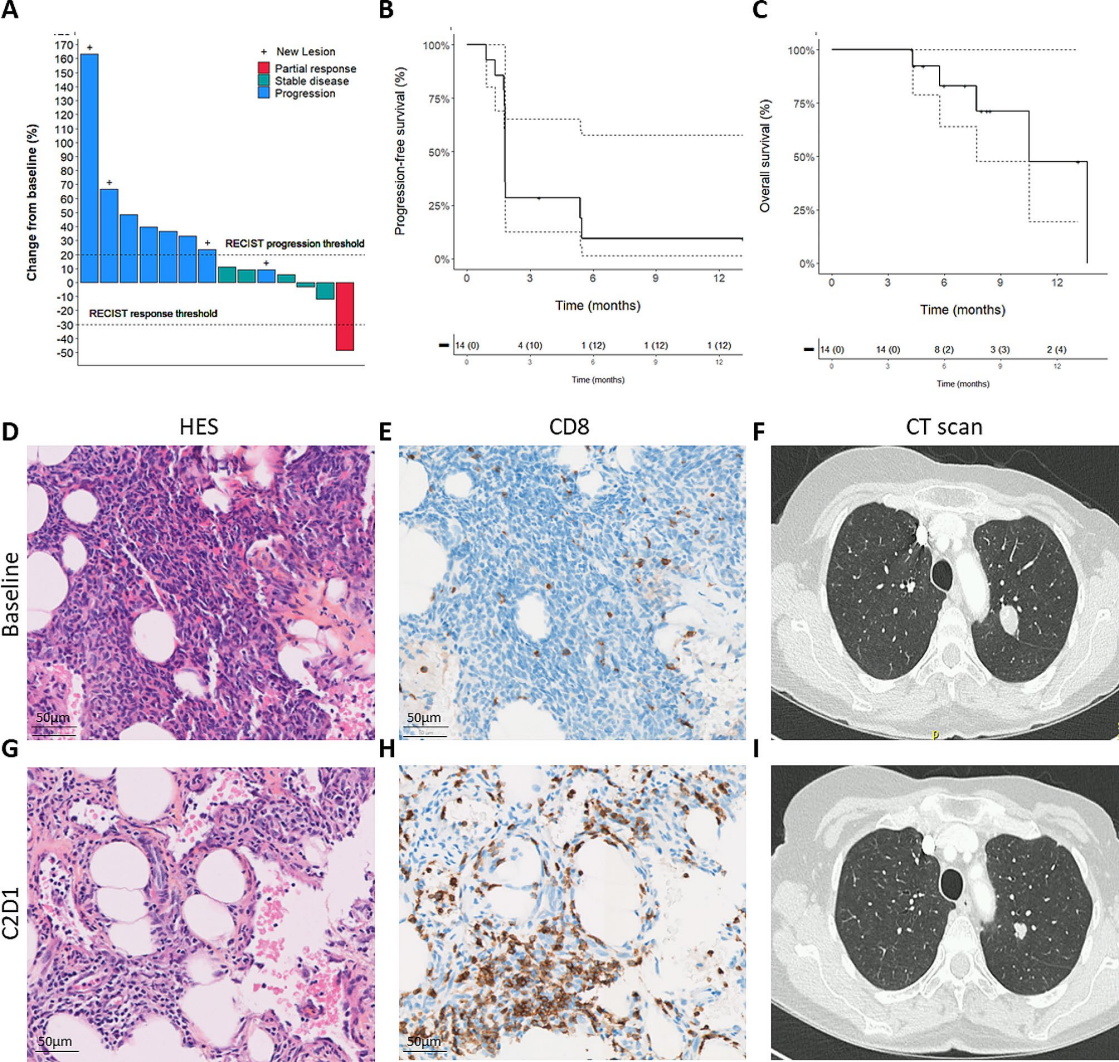
Efficacy of JX-594 combined with low-dose cyclophosphamide and avelumab in patients with advanced “cold” sarcomas. **(A)** Waterfall plot of the maximum change in tumor size in 14 patients with sarcomas and eligible for efficacy analysis. **(B-C)** Kaplan–Meier curves associated with Progression-free survival **(B)** and overall survival **(C)**. **(D-I)** Illustrations of tumor HES staining **(D-G)**, tumor CD8 staining **(E-H)** and Computed Tomography scan **(F-I)** obtained from the patient with partial response at baseline **(D-E-F)** and C2D1**(G-H-I)**

After a median follow-up of 8.2 months (95% CI 4.8–13.1, reverse Kaplan-Meier), 13 patients discontinued treatment. Discontinuation was related to disease progression in 12 cases and to patient’s decision in one case. Among the 14 patients assessable for the efficacy analysis (Supplementary Fig. 1), one was progression-free at 6 months indicating that the first stage of the Simon’s design was not satisfied. Best response as per RECIST criteria was partial response for one patient (1 angiosarcoma), stable disease for 5 patients (2 leiomyosarcoma, 1 angiosarcoma, 1 epithelioid sarcoma, 1 low-grade fibromyxoid sarcoma) and progressive disease for 8 patients (4 leiomyosarcomas, 1 dedifferentiated liposarcoma, 1 endometrial stromal sarcoma, 1 myxoid liposarcoma, 1 undifferentiated pleomorphic sarcoma) (Fig. 1A).

Five patients died during the study. Death was due to disease progression in all the cases. Median progression-free survival and overall survival were 1.8 months (95% CI 1.7–3.6) and 10.5 months (95% CI 5.7–13.6) respectively (Fig. 1B and C).

All the patients were included in the safety analysis. At the time of analysis, 50 cycles of JX-594, avelumab and metronomic CP had been administered, with a median of 2 cycles per patient (range 1–9). The most observed toxicities were grade 1 fever and grade 1 fatigue (Supplementary Table 1). Grade 3 toxicities were rare and included three cases of grade 3 fever. No grade 4 toxicity was observed.

To assess the systemic immune response elicited by oncolytic virotherapy, we analyzed the percentage of CD8 + tumor-infiltrating lymphocytes (TILs) in 11 paired biopsy specimens of a non-injected lesion, collected at baseline and on Day 1 of Cycle 2, as a potential pharmacodynamic biomarker. Among these patients, 10 exhibited an increase in CD8 + TILs during therapy compared to baseline. A significant elevation in the percentage of CD8 + TILs was noted, with an average increase of 3.9 times over baseline levels in on-therapy specimens (ranging from 0.7 to 11.8 fold change, *P* = 0.0029) (Supplementary Fig. 2). Notably, the three patients demonstrating the most substantial increases were diagnosed with undifferentiated pleomorphic sarcoma (UPS), angiosarcoma, and leiomyosarcoma, respectively. Specifically, the patient with an objective response (diagnosed with angiosarcoma) exhibited the most pronounced augmentation in CD8 + TIL percentage.

We also explored blood-based immune biomarkers by conducted a proteomic profiling of plasma samples collected at baseline and during treatment. While minimal alterations in the plasma proteome were observed following the initial injection of JX-594 compared to baseline, a significant upregulation of proteins associated with T cell immune responses (e.g., ICOS-LG) and T cell cytotoxicity (e.g., granzymes) was detected when comparing plasma samples from C2D1 (post first avelumab and second JX-594 injection) to baseline (Supplementary Fig. 3).

## Discussion

In this manuscript, we present the findings from a phase II study exploring the effects of a genetically engineered oncolytic virus on the tumor microenvironment (TME) in patients with TLS-negative soft tissue sarcoma (STS). This research represents a significant step forward in the field, offering a novel perspective on the potential of oncolytic virotherapy to remodel the TME, thereby enhancing tumor susceptibility to immune-mediated tumor destruction.

Despite the modest clinical activity observed in our cohort, these findings contribute significantly to the evolving landscape of oncolytic virotherapy in STS. A contrasting point of interest emerges when considering the recent study, where intra-tumoral administration of talimogene laherparepvec (T-VEC), an oncolytic immunotherapy derived from a modified human herpes simplex virus type 1, was combined with pembrolizumab in patients with advanced STS. The objective response rate was significantly higher than in our study. However, among the responders with available pre-treatment tumor tissue, 75% exhibited strong tumor-infiltrating lymphocyte (TIL) infiltration at baseline [13]. This finding suggests a potentially more ‘inflamed’ TME in their patient cohort compared to ours, characterized by a ‘desert’ phenotype with no tertiary lymphoid structures and low CD8 + T cell density.

In-depth analysis of sequential tumor biopsies and plasma proteomics revealed that treatment with JX-594 leads to significant changes in the TME, particularly by fostering T cell accumulation. A strength of our study is that the sequential biopsies were obtained from non-injected lesions to confirm the systemic immunological impact of the intratumoral administration of JX-594. Our results align with preclinical findings and suggests that JX-594 has the potential to convert immunologically ‘cold’ tumors into more immunogenic ones, thereby enhancing their sensitivity to immune checkpoint inhibitors (ICIs) [14]. Notably, the patient who achieved an objective response in our study also showed the most pronounced increase in CD8 + T cell density, indicating a possible direct correlation between TME modification and therapeutic response.

However, our study’s limited sample size precluded a comprehensive subgroup analysis based on STS histological subtypes. Recognizing this limitation, future research should be adequately powered to thoroughly investigate the impact of oncolytic virotherapy on the TME across different STS subtypes. Such studies are essential to unravel the complex interactions between oncolytic viruses, the TME, and tumor histology. The insights gained could be instrumental in developing more effective, personalized therapies for patients with advanced STS, ultimately improving clinical outcomes in this heterogeneous and challenging patient population.

### Electronic supplementary material

Below is the link to the electronic supplementary material.

Supplementary Material 1

## Data Availability

No datasets were generated or analysed during the current study.

## Declarations

### AI

research grant (ASTRAZENECA, BAYER, BMS, IPSEN, MERCK, MSD, NOVARTIS, PARTHENON, ROCHE); advisory board (ASTRAZENECA, BAYER, BMS, DAIICHI SANKYO, IPSEN, MERCK, MSD, NOVARTIS, PARTHENON, ROCHE).

### JPG, AB

employees of Explicyte. The other authors have nothing to declare.

## Abbreviations

CCT: Conventional Cytotoxic Chemotherapy
C1D15: Cycle 1 Day 15
C2D1: Cycle 2 Day 1
CP: Cyclophosphamide
DAB: Diaminobenzidine
ECOG: Eastern Cooperative Oncology Group
FFPE: Formalin-Fixed Paraffin-Embedded
GM: CSF-Granulocyte-Macrophage Colony-Stimulating Factor
ICIs: Immune Checkpoint Inhibitors
IHC: Immunohistochemistry
IT: Intra-tumoral
MC: Metronomic Chemotherapy
OS: Overall Survival
OV: Oncolytic Viruses
PFS: Progression-Free Survival
pRB/E2F: Retinoblastoma/E2F
RECIST: Response Evaluation Criteria in Solid Tumours
SIC: Sarcoma Immune Classes
STS: Soft-Tissue Sarcomas
TILs: Tumor-Infiltrating Lymphocytes
TK: Thymidine Kinase
TLS: Tertiary Lymphoid Structures
