# Supplementary material for: Reshaping the tumor microenvironment of cold soft-tissue sarcomas with oncolytic viral therapy: a phase 2 trial of intratumoral JX-594 combined with avelumab and low-dose cyclophosphamide
Source: Mol Cancer. 2024 Feb 20;23:38. doi: 10.1186/s12943-024-01946-8 (PMC10877825; doi:10.1186/s12943-024-01946-8)
Supplement: Supplementary file 1 — Supplementary Material 1 [file 12943_2024_1946_MOESM1_ESM.docx]

**SUPPLEMENTARY TABLES**

**Supplementary Table 1: Number of patients presenting at least one adverse effect related to Cyclophosphamide and/or JX-594 and/or avelumab by AE term, SOC and grade in Arm 2 (n=14)**

| **Maximum intensity** | | | | | | |
| --- | --- | --- | --- | --- | --- | --- |
| **Grade 1/2** | | | **Grade 3** | | **Grade 4** | |
| **N** |  | **%** | **N** | **%** | **N** | **%** |
| Anemia | 5 | 33.3 | . | . | . | . |
| Hyperthyroidism | 2 | 13.3 | . | . | . | . |
| Abdominal Pain | 3 | 20.0 | . | . | . | . |
| Diarrhea | 2 | 13.3 | . | . | . | . |
| Mucositis oral | 5 | 33.3 | . | . | . | . |
| Nausea | 9 | 60.0 | . | . | . | . |
| Vomiting | 7 | 46.6 | . | . | . | . |
| Chills | 11 | 73.3 | . | . | . | . |
| Fatigue | 12 | 80.0 | 3 | 60.0 | . | . |
| Fever | 14 | 90.3 | 2 | 40.0 | . | . |
| Rash pustular | 8 | 53.3 | . | . | . | . |
| Infusion related reaction | 3 | 20 | 1 | 6.7 |  |  |
| Alanine aminotransferase increased | 1 | 6.7 | . | . | . | . |
| Alkaline phosphatase increased | 1 | 6.7 | 1 | 6.7 | . | . |
| Lymphopenia | 1 | 6.7 | 1 | 6.7 | . | . |
| Headache | 4 | 26.7 | . | . | . | . |
| Hypertension | 3 | 20 | 1 | 6.7 | . |  |
| Hypotension | 6 | 40 | 2 | 13.3 |  |  |
| Pruritus | 2 | 13.3 | . | . | . | . |
| Dry skin | 2 | 13.3 | . | . | . | . |

**SUPPLEMENTARY FIGURES**

**
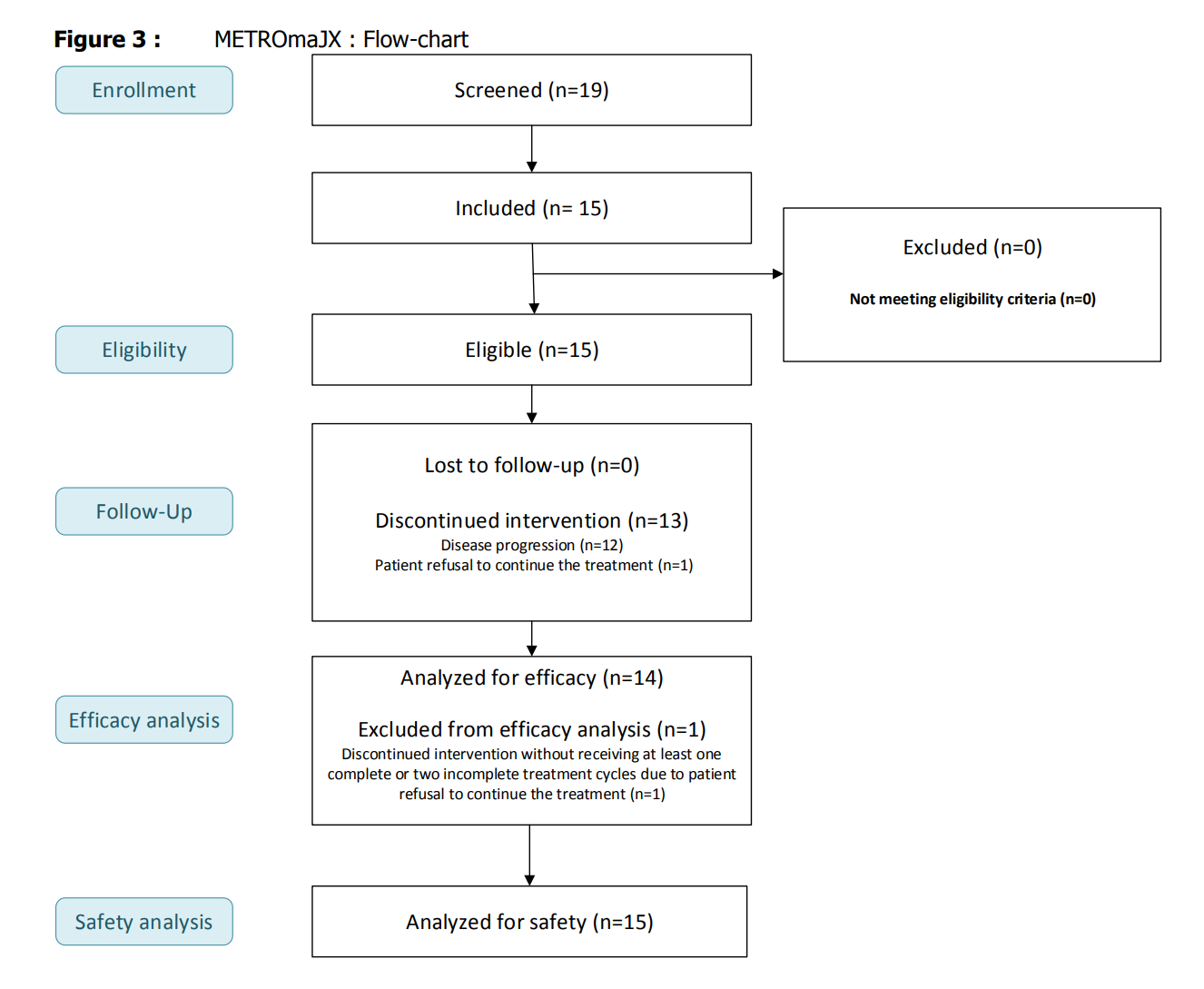
**

**Supplementary Figure 1. Flow chart of the METROMAJX study**

**
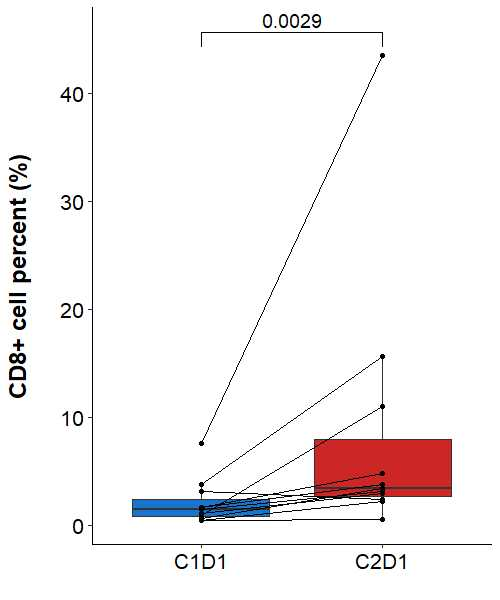
**

**Supplementary Figure 2.** CD8+ TIL percentage as a measure of pharmacodynamic activity in the METROMAJX study (n=11)


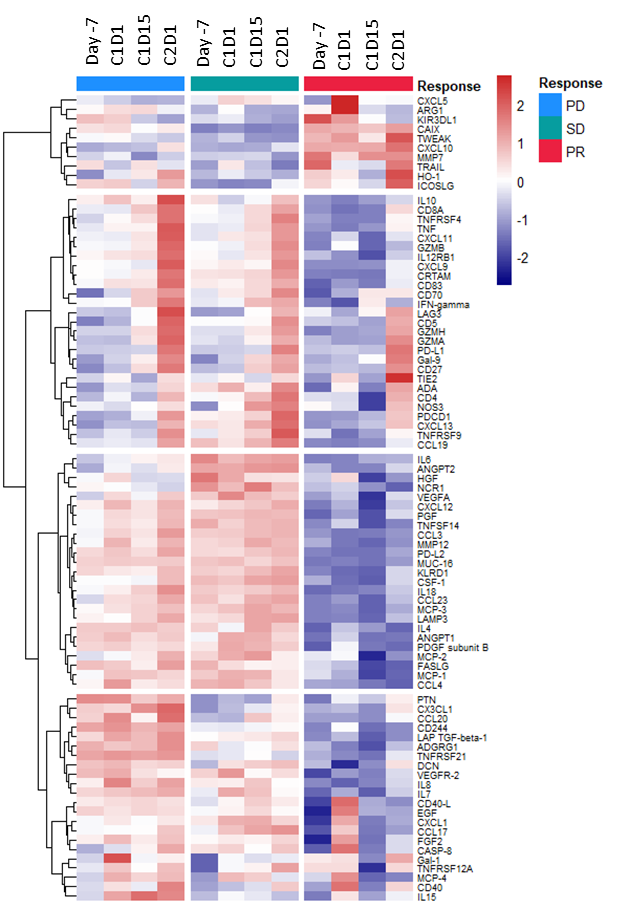


**Supplementary Figure 3**. Evolution of the plasma proteome as a measure of pharmacodynamic activity in the METROMAJX study (n=11).
